# From probiotic depletion to inflammatory cascade: multi-omics reveals the temporal progression of sleep deprivation-induced gut-liver axis disruption in mice

**DOI:** 10.3389/fmicb.2026.1846136

**Published:** 2026-06-05

**Authors:** Ming-Kun Dai, Xiao Kuang, Yi-Fei Wang, Xiao-Xiao Liu, Yong-Fan Jin, Yi-Ying Li, Wen-Lin Tai

**Affiliations:** 1 Department of Clinical Laboratory, The Second Affiliated Hospital of Kunming Medical University, Kunming, Yunnan, China; 2 Department of Burns Surgery, The Second Affiliated Hospital of Kunming Medical University, Kunming, Yunnan, China

**Keywords:** arachidonic acid metabolism, gut microbiota, gut-liver axis, hepatic metabolomics, sleep deprivation

## Abstract

**Introduction:**

Sleep deprivation (SD) is increasingly recognized as a risk factor for metabolic and hepatic disorders, yet the temporal dynamics linking gut dysbiosis to liver injury remain poorly characterized.

**Methods:**

In this study, we established a platform-based SD model in a total of 24 C57BL/6J mice (*n* = 4 per group per weekly time point) over 3 weeks and systematically investigated the weekly progression of gut microbiome alterations, hepatic metabolite profiles, and liver injury markers using Accu16S^®^ absolute quantification sequencing and untargeted LC-MS/MS metabolomics.

**Results:**

Our results revealed a temporally ordered three-stage cascade of gut-liver axis disruption. In week 1, core probiotics including *Bifidobacterium* and *Dubosiella* declined concurrently with protective metabolites such as tryptamine and glycocholic acid, alongside perturbation of primary bile acid biosynthesis. In week 2, concurrent enrichment of a compensatory Lactobacillaceae member (*Ligilactobacillus*) and an opportunistic pathogen (*Streptococcus*) was accompanied by reduced microbiota–metabolite correlation density, coinciding with peak serum ALT/AST levels and metabolic pathway remodeling involving nucleotide sugar and tryptophan metabolism. By week 3, synchronous decline of *Lactobacillus*, *Bifidobacterium*, and *Dubosiella* coincided with full-scale activation of arachidonic acid metabolism and inflammatory immune pathways, accompanied by elevated serum LPS levels suggestive of intestinal barrier perturbation. Correlation analyses revealed that the progressive depletion of beneficial bacteria was associated with the sequential loss of protective metabolites, reorganization of the metabolic network, and inflammatory mediator accumulation, although these relationships remain correlational and require causal validation.

**Discussion:**

These findings describe a “protective loss–homeostatic decoupling–inflammatory signature” descriptive model. Because these findings are based on correlation analyses in a mouse model without causal validation, further mechanistic and interventional studies are required before any clinical implications can be drawn.

## 1 Introduction

Sleep is a fundamental biological process essential for maintaining physiological homeostasis across multiple organ systems. Epidemiological studies have consistently demonstrated that chronic sleep insufficiency, affecting approximately one-third of the global adult population, is associated with increased risks of metabolic syndrome, cardiovascular disease, and hepatic dysfunction (Chattu et al., 2018). Despite growing recognition of the consequences of SD, the mechanistic pathways linking sleep loss to organ damage–particularly liver injury–remain incompletely understood.

The liver, as a central hub of metabolic regulation, is highly susceptible to perturbations in systemic homeostasis. Prolonged sleep restriction has been shown to disrupt lipid metabolism, alter bile acid composition, and activate inflammatory signaling cascades in the liver (Aho et al., 2016). Studies have revealed that SD leads to early translocation of intestinal bacteria to otherwise sterile tissues, triggering persistent endogenous infection and chronic immune activation (Everson and Toth, 2000). However, how these disturbances evolve over time has not been systematically characterized.

The gut-liver axis has emerged as a critical pathway mediating the systemic effects of SD. The intestinal microbiota, comprising trillions of microorganisms, plays an indispensable role in nutrient metabolism, bile acid biotransformation, immune modulation, and maintenance of the intestinal epithelial barrier (Tripathi et al., 2018). Disruptions in the gut microbiome–termed dysbiosis–can increase intestinal permeability, facilitate translocation of microbial products such as lipopolysaccharide (LPS) into the portal circulation, and subsequently trigger hepatic inflammation through toll-like receptor signaling pathways (Albillos et al., 2020). Several studies have demonstrated that SD significantly alters gut microbial composition in both rodent models and human subjects. Poroyko et al. (2016) reported that sleep fragmentation in mice induced gut dysbiosis characterized by increased abundance of Firmicutes and enhanced intestinal permeability. Benedict et al. (2016) further showed that even short-term partial SD in humans altered the Firmicutes-to-Bacteroidetes ratio, a hallmark of metabolic dysbiosis.

The bidirectional communication between the gut microbiota and hepatic metabolism is largely mediated through microbial metabolites. Short-chain fatty acids (SCFAs), tryptophan catabolites, secondary bile acids, and other microbiota-derived metabolites serve as signaling molecules that regulate hepatic lipid metabolism, gluconeogenesis, and inflammatory responses (Canfora et al., 2019). Tryptamine, a microbial metabolite of tryptophan, has been demonstrated to activate the aryl hydrocarbon receptor (AhR) signaling pathway, thereby suppressing liver inflammation and enhancing intestinal barrier integrity (Roager and Licht, 2018). Similarly, bile acid metabolism–regulated through the microbiota-dependent farnesoid X receptor (FXR) and Takeda G protein-coupled receptor 5 (TGR5) signaling axes–is crucial for maintaining hepatic metabolic homeostasis (Wahlström et al., 2016). Despite these mechanistic insights, the temporal interplay between SD-induced gut dysbiosis and hepatic metabolic remains largely unexplored. Metabolomics, particularly untargeted liquid chromatography-tandem mass spectrometry (LC-MS/MS), provides a powerful tool for comprehensively profiling metabolic alterations in biological systems. Combined with 16S rRNA gene sequencing of the gut microbiome, integrative multi-omics approaches enable the identification of microbiota-metabolite co-variation networks that may underlie disease pathogenesis. However, few studies have employed longitudinal, time-resolved multi-omics strategies to dissect the dynamic progression of SD-induced gut-liver axis perturbations.

In this study, we established a platform-based SD model in C57BL/6J mice over a 3-weeks period and systematically investigated the weekly progression of gut microbiome composition, hepatic metabolite profiles, and liver injury markers. By integrating Accu16S^®^ absolute quantification sequencing with untargeted metabolomics and correlation analyses, we aimed to describe the sequential pattern through which SD-associated gut dysbiosis and hepatic metabolic alterations evolve over time. We hypothesized that SD is associated with a temporally ordered progression from protective metabolite depletion and bile acid dysregulation (early phase), through metabolic network remodeling and reduced microbiota–metabolite correlation density (intermediate phase), to enrichment of arachidonic acid metabolism and inflammatory immune pathways (late phase), thereby providing a descriptive framework for the gut microbiota–metabolite axis in SD-associated hepatic pathology that warrants subsequent causal validation.

## 2 Materials and methods

### 2.1 Experimental design

A total of 24 Male C57BL/6J mice aged 6–8 weeks (purchased from Beijing SPF Biotechnology Co., Ltd.) were housed under a 12/12-h light/dark cycle with *ad libitum* access to food and water. Mice were randomly assigned to two groups using a computer-generated randomization table. The platform-based protocol used here is a modified version of the multiple-platform-over-water paradigm. To control for the physical-restraint and stress-related confounders inherent to the platform-based paradigm, the control group (CON) was housed under matched conditions: each control mouse was placed on a single 5 cm diameter cylindrical platform positioned in an identical water tank (32 × 21 × 16 cm) filled with water to a level of 1 cm below the platform surface. The larger platform diameter permits normal sleep posture and brief limb relaxation while imposing comparable handling, water-tank exposure, social-housing arrangement, and circadian timing as the SD group, thereby isolating sleep loss as the principal variable differing between groups. The SD group underwent platform-based SD on platforms that were 2 cm in diameter and placed in identical water tanks (32 × 21 × 16 cm) filled with water to a level of 1 cm below the platform surface. Four mice from the same home cage were placed in each SD water tank, with control animals housed in matched cages on the larger platforms, for 6 h per day with *ad libitum* access to food and water. SD commenced at Zeitgeber time (ZT) 1 (corresponding to 8:00 AM Eastern Standard Time) and concluded at ZT7 on the same day. Following euthanasia by isoflurane overdose, blood, colonic fecal contents, and liver tissues were collected from both groups at ZT8 at weekly intervals (four mice per group per week, over a period of 3 weeks). Sample sizes for each group were determined based on our prior experience and sample sizes used in comparable experiments and analyses in published studies. To enable continuous behavioral monitoring of sleep deprivation across the experiment, all water tanks were filmed throughout each daily 6-h session by overhead night-vision infrared cameras under low ambient light, allowing observation of activity patterns, postural adjustments, and platform–water interactions in both groups.

### 2.2 Oil Red O staining

Lipid accumulation in liver tissues was assessed using Oil Red O staining according to a standard protocol. Briefly, liver tissue sections were fixed with 4% paraformaldehyde for 15 min, washed with 60% isopropanol for 1 min, and then stained with Oil Red O working solution for 10 min. Sections were subsequently rinsed with 60% isopropanol and distilled water prior to examination under light microscopy.

### 2.3 TUNEL assay

Paraffin-embedded liver tissue sections were prepared for the detection of apoptotic cells using terminal deoxynucleotidyl transferase dUTP nick end labeling (TUNEL) with a commercially available kit (G1507, Servicebio, China) according to the manufacturer’s instructions. TUNEL-positive cells were visualized and quantified under light microscopy.

### 2.4 Enzyme-linked immunosorbent assay (ELISA)

Mouse serum was collected, and LPS levels were measured using a commercially available ELISA kit (SEB526Ge, Cloud-Clone Corp., USA). All assays were performed in accordance with the manufacturer’s instructions.

### 2.5 Serum biochemical analysis

Whole blood obtained from mice was allowed to stand at room temperature for 1 h and subsequently centrifuged at 5,000 rpm for 15 min at 4 °C to separate the serum. The resulting supernatant was diluted fivefold with physiological saline. Serum levels of total cholesterol (TC), total triglycerides (TG), aspartate aminotransferase (AST), and alanine aminotransferase (ALT) were determined using a fully automated biochemistry analyzer (Chemray 800, Shenzhen Rayto Life Science, China).

### 2.6 Histology and histopathology

Liver samples were fixed in 4% paraformaldehyde and embedded in paraffin wax. Sections were stained with hematoxylin and eosin (H&E) to assess histological damage using light microscopy.

### 2.7 Metabolomic profiling

Untargeted metabolomic analysis was performed by Shanghai G&C Biotechnology Co., Ltd. Sample preparation, instrument acquisition, and data preprocessing were performed as follows. Reagents and standards: LC-MS grade acetonitrile and methanol were purchased from Fisher Scientific (Loughborough, UK), chloroform from Sinopharm (Shanghai, China), formic acid from TCI (Shanghai, China), and ammonium formate from Sigma-Aldrich (Shanghai, China). Ultrapure water was generated using a Milli-Q system (Millipore, Bedford, USA). 2-Amino-3-(2-chloro-phenyl)-propionic acid (2-chloro-L-phenylalanine; Aladdin, Shanghai, China) was used as the internal standard. Sample preparation: an accurately weighed aliquot of liver tissue was transferred to a 2 mL centrifuge tube, 1000 μL of tissue extraction solvent [75% (9:1, v/v methanol:chloroform):25% H2O] was added together with two pre-chilled steel balls, and samples were homogenized in a tissue grinder (Meibi MB-96) at 50 Hz for 60 s, with the homogenization step repeated twice. Homogenates were sonicated for 15 min at room temperature, then placed on ice for 15 min, and centrifuged at 12,000 rpm at 4 °C for 10 min in a high-speed refrigerated centrifuge (Xiangyi H1850-R). The full supernatant was transferred to a new tube and evaporated to dryness in a centrifugal vacuum concentrator (Eppendorf 5305). Dried extracts were reconstituted in 400 μL of 50% acetonitrile containing 4 ppm 2-chloro-L-phenylalanine, filtered through a 0.22 μm PTFE membrane (Jinteng, Tianjin, China), and transferred to autosampler vials for LC-MS analysis. Pooled QC samples were prepared by combining equal aliquots from all study samples and were processed and injected alongside the study samples to monitor instrument stability and to enable retention-time and signal-intensity correction. Liquid chromatography: separations were performed on a Thermo Vanquish UHPLC system (Thermo Fisher Scientific, USA) using an ACQUITY UPLC HSS T3 column (2.1 mm × 100 mm, 1.8 μm; Waters, Milford, MA, USA) maintained at 40 °C, with a flow rate of 0.3 mL/min and an injection volume of 5 μL. For positive-ion mode (ESI+), the mobile phases were 0.1% formic acid in acetonitrile (B2) and 0.1% formic acid in water (A2); for negative-ion mode (ESI−), the mobile phases were acetonitrile (B3) and 5 mM ammonium formate in water (A3). The same gradient program was applied in both ionization modes: 0–1 min, 10% B; 1–5 min, 10%–98% B; 5–6.5 min, 98% B; 6.5–6.6 min, 98%–10% B; 6.6–8 min, 10% B. Mass spectrometry: detection was performed on a Thermo Orbitrap Exploris 120 mass spectrometer (Thermo Fisher Scientific, USA) equipped with an electrospray ionization source operated in both positive and negative modes (full MS–ddMS2 acquisition). Source parameters were: spray voltage 3.50 kV (ESI+) and −2.50 kV (ESI−); sheath gas 40 arb; auxiliary gas 10 arb; capillary temperature 325 °C. MS1 was acquired at a resolving power of 60,000 FWHM over m/z 100–1000, with the top 4 ions per cycle selected for HCD fragmentation at 30% normalized collision energy and an MS/MS resolving power of 15,000 FWHM; dynamic exclusion was applied to suppress redundant MS/MS triggering. Data preprocessing: raw mass spectrometry files were converted to mzXML format using MSConvert from the ProteoWizard suite (v3.0.8789), and peak detection, peak filtering, and peak alignment were performed in R using the XCMS package [v3.12.0; method = “centWave”, ppm = 15, peakwidth = c(5, 30), bw = 2, mzwid = 0.015, mzdiff = 0.01] to generate a metabolite quantification matrix. Signal intensities were corrected using QC-based correction to remove systematic technical variation, and features with a relative standard deviation (RSD) > 30% across QC injections were filtered out prior to downstream statistical analysis.

### 2.8 Gut microbiome assessment and processing of 16S rDNA sequencing data

Intestinal contents were collected on the end of each weekly intervention period, after an overnight fast without food or water. Following anesthesia and laparotomy, intestinal contents were collected from the mid-colon for Accu16S^®^ analysis (precise 16S absolute quantification sequencing), which was performed by Shanghai G&C Bio-technology Co., Ltd. Briefly, total genomic DNA was extracted from samples using the FastDNA^®^ SPIN Soil DNA Extraction Kit (MP Biomedicals, USA) according to the manufacturer’s protocol. DNA concentration and purity were determined using a Nanodrop 2000 spectrophotometer and a Qubit 3.0 fluorometer. Multiple synthetic spike-in sequences were designed, which retained the conserved regions of the native 16S rRNA gene but contained variable regions replaced with random sequences with a GC content of approximately 40%. Spike-in mixtures of known gradient copy numbers were added to the sample DNA at appropriate proportions. The V3–V4 hypervariable regions of the 16S rRNA gene and the spike-in sequences were amplified using primers 341F and 805R, and the amplification products were sequenced on the BGI G99 plat-form using paired-end 250 bp reads. Raw sequencing reads were processed using QIIME2 (version 2023.2) (Bolyen et al., 2019). Adapter and primer sequences were first removed using the cutadapt plugin (trim-paired –p-error-rate 0.15, p-trunc-len-f/r = 220, p-trim-left-f/r = 0). Subsequently, raw data were denoised, merged, and subjected to chimera removal using the DADA2 plugin to generate high-quality amplicon sequence variant (ASV) feature tables and representative sequences (denoise-paired MaxEE = 2, truncQ = 2, p-chimera-method = consensus, p-min-fold-parent-over-abundance = 1.0) (Callahan et al., 2016). ASVs were taxonomically classified using a pre-trained classifier (classify-sklearn –p-confidence 0.8), with a confidence threshold of 0.8 for 16S rRNA gene classification and 0.6 for ITS region classification. Spike-in sequences were identified and their read counts were used to construct standard curves. Species copy numbers per unit sample were calculated based on the sequencing template DNA quantity, total extracted DNA yield, and the amount of sample used for DNA extraction.

### 2.9 Statistical analysis

Statistical analysis was performed with GraphPad Prism 10.0 software. All of the values in the present study are presented as the means ± SD. Unpaired Student’s *t*-test was used for two-group comparisons of biochemical and phenotypic indicators, and one-way ANOVA with Tukey’s *post-hoc* test was used when more than two groups were compared. For the high-dimensional metabolomics and microbiome datasets, raw *p*-values were adjusted for multiple testing using the Benjamini–Hochberg false discovery rate (FDR) procedure; adjusted *p*-values (*q*-values) < 0.05 were considered statistically significant, and differential metabolites were additionally required to have a variable importance in projection score > 1 from PLS-DA modeling. Microbiota–metabolite Pearson correlations were similarly FDR-adjusted across the full set of comparisons performed at each time point. Significant *p*-values from univariate comparisons are denoted by *p-value < 0.05, **p-value < 0.01, ***p-value < 0.001, ns represents no significance.

## 3 Results

### 3.1 SD altered body weight, liver weight, and liver-to-body weight ratio in mice

**FIGURE 1:**
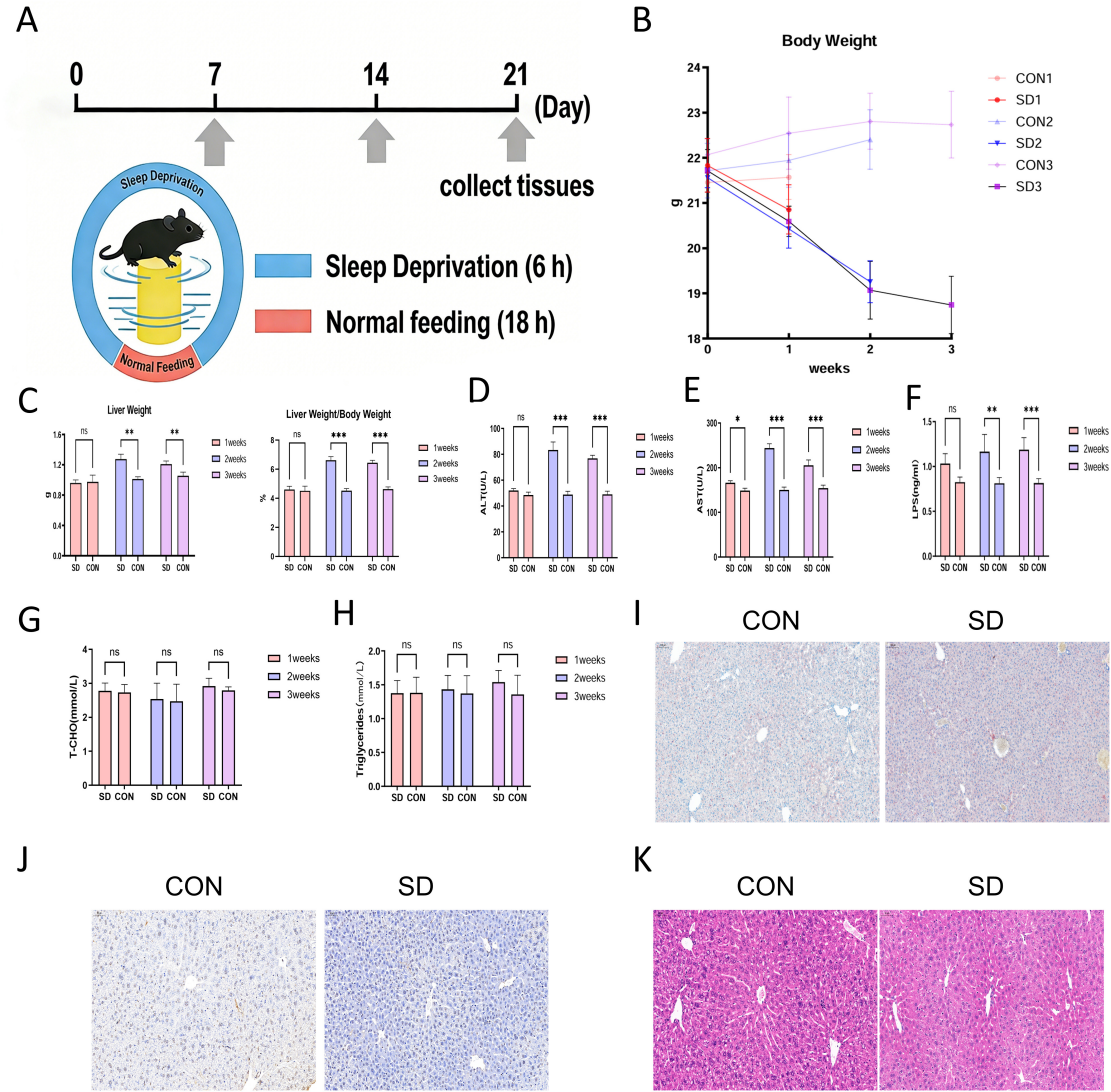
Sleep deprivation is associated with biochemical and metabolic alterations in mice. **(A)** Schematic diagram of the experimental design showing the platform-based SD model over 3 weeks. **(B)** Body weight changes in control (CON) and SD groups across the 3-weeks period, plotted as mean ± SD for each time point. **(C)** Liver weight and liver-to-body weight ratio in CON and SD groups at weeks 1, 2, and 3. **(D,E)** Serum levels of ALT and AST in CON and SD groups at weeks 1, 2, and 3. **(F)** Serum LPS levels in CON and SD groups at weeks 1, 2, and 3. **(G)** Serum TC levels in CON and SD groups. **(H)** Serum TG levels in CON and SD groups. **(I)** Representative Oil Red O staining images of liver tissue sections from CON and SD groups. **(J)** Representative TUNEL fluorescence assay images of liver tissue sections from CON and SD groups. **(K)** Representative H&E staining images of liver tissue sections from CON and SD groups. Data are presented as mean ± SD (*n* = 4 per group per time point). *P < 0.05, **P < 0.01, ***P < 0.001 vs. CON group; ns, not significant.

During the 3-weeks SD period, all mice survived and remained in good general condition regardless of group assignment (Figure 1A). Compared with the control group, the body weight of SD mice was significantly reduced during weeks 1 and 2, with a diminished but persistent downward trend in week 3 (Figure 1B). These findings indicate that SD reduces body weight in mice, consistent with previous reports (Đukanović et al., 2022). Continuous night-vision infrared recording over each daily 6-h session further showed that SD-group mice exhibited disrupted activity patterns relative to the large-platform control group, including disturbed activity cycles, shorter cumulative rest durations, and frequent postural adjustments associated with the small-platform fall-off reflex. Although these observations are qualitative and complementary to–rather than a substitute for–electroencephalography-based sleep staging, they provide direct in-cage behavioral evidence consistent with effective sleep deprivation in the SD group. Liver weight in the SD group showed no significant change in week 1, increased significantly in week 2, and slightly decreased in week 3 while remaining above week 1 levels, suggesting that week 2 may represent the peak of hepatic injury with partial but incomplete recovery by week 3. Changes in the liver-to-body weight ratio were even more pronounced, with the magnitude of increase exceeding that of absolute liver weight gain. The trend was similar to that of liver weight, with a significant increase in week 2 followed by a slight decrease in week 3 that nonetheless remained above week 1 values (Figure 1C).

### 3.2 SD induced hepatic metabolic alterations

We first assessed serum levels of ALT and AST, two widely used biochemical biomarkers of hepatic injury. Serum ALT and AST levels in the SD group were mildly elevated during weeks 2 and 3, with the increases peaking at week 2 (Figures 1D, E). While these changes are consistent with subclinical hepatocellular stress, ALT/AST elevations alone are not specific to overt hepatic injury. No significant difference in serum LPS levels was observed between groups at week 1, whereas LPS levels were significantly increased in the SD group at weeks 2 and 3, suggesting that sustained SD may be associated with a low-grade systemic inflammatory signal (Figure 1F).

Total cholesterol levels showed no significant differences between groups, and TG levels were elevated but did not reach statistical significance (Figures 1G,H), indicating that systemic lipid metabolic parameters were not markedly altered over this timeframe. Oil Red O staining of liver tissue (Figure 1I) revealed no obvious increase in microvesicular lipid droplet accumulation in hepatocytes in the SD group compared with controls, as assessed by blinded qualitative scoring of three randomly selected high-power fields per section across all animals. Histopathological analysis by H&E staining and TUNEL fluorescence assay (Figures 1J,K) likewise did not reveal overt hepatic congestion, architectural disruption, or widespread apoptosis. Taken together, the biochemical (ALT, AST, LPS) and histological findings indicate that 3 weeks of SD produced biochemical alterations consistent with early, subclinical hepatocellular stress rather than established structural or steatotic liver injury.

### 3.3 Temporal dynamics of gut microbiota composition

**FIGURE 2:**
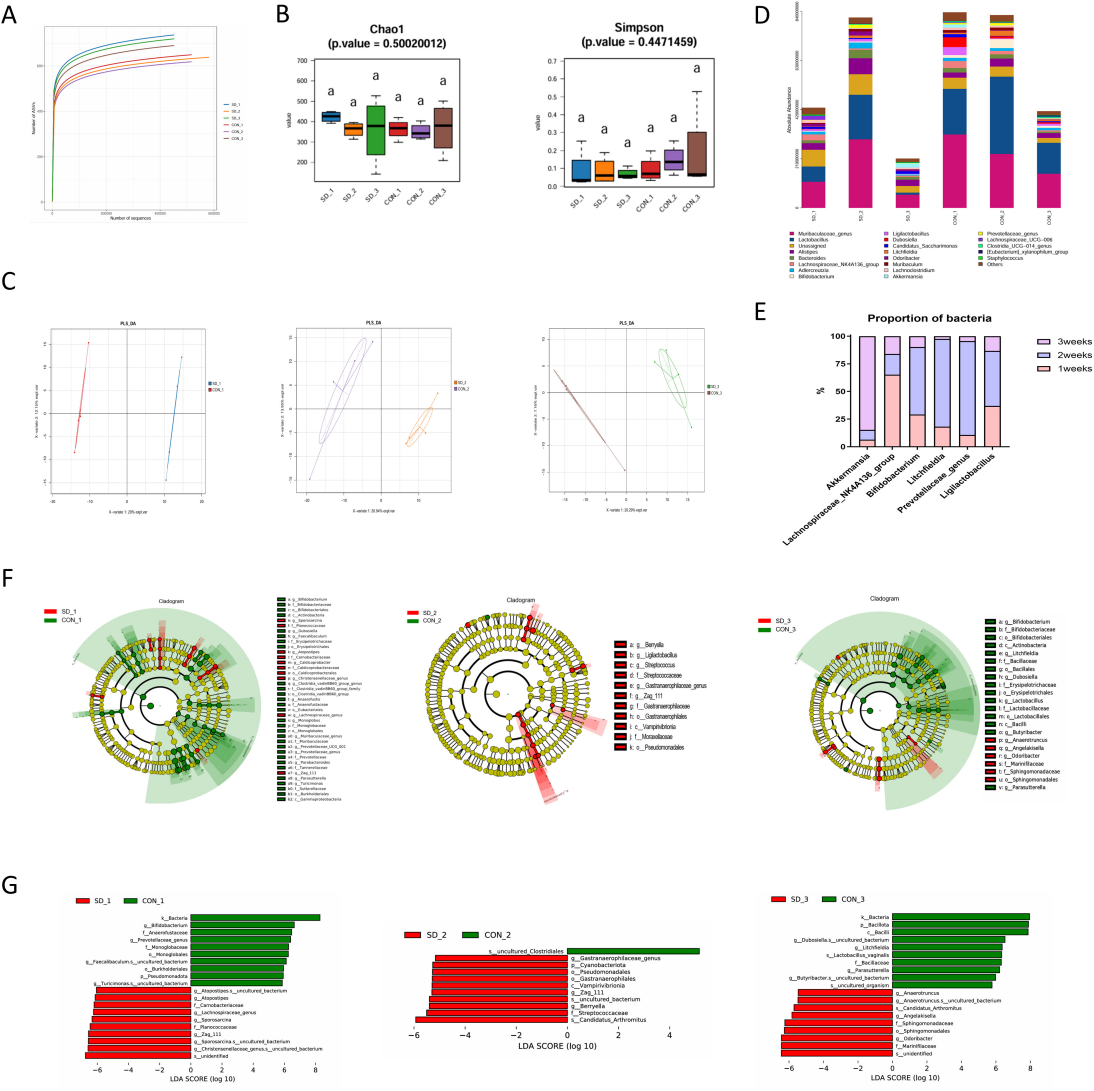
Temporal pattern of gut microbiota composition in sleep-deprived mice. Panels are organized into three thematic blocks for readability: (i) overall community structure **(A–C)**, (ii) between-group differential taxa at each weekly time point **(D,F,G)**, and (iii) within-SD-group temporal trajectories **(E)**. **(A)** Species-level absolute abundance profiles of gut microbiota in CON and SD groups across weeks 1, 2, and 3. **(B)** Alpha diversity analysis of gut microbiota using Chao1 index and Simpson index in CON and SD groups at each time point. **(C)** PLS-DA score plots showing beta-diversity separation between CON and SD groups at weeks 1, 2, and 3. **(D)** Bar charts displaying differentially abundant bacterial genera at the species level between CON and SD groups at weeks 1, 2, and 3. **(E)** Temporal pattern of individual bacterial genera within the SD group across the 3-weeks period, showing genera that peaked at week 2, exhibited sustained increases, or showed continuous decline. **(F)** LEfSe linear discriminant analysis (LDA) scores for differentially abundant taxa between CON and SD groups at weeks 1, 2, and 3 (LDA score threshold = 5). **(G)** Cladograms illustrating the taxonomic distribution of differentially abundant bacterial taxa identified by LEfSe analysis at weeks 1, 2, and 3. Data are presented as mean ± SD (*n* = 4 per group per time point).

We first sought to determine whether SD affects the overall gut microbiota. To this end, we compiled and analyzed microbiota profiling results across different time points. Species-level abundance analysis revealed that both groups exhibited sufficient species richness and high abundance to support in-depth characterization of the gut microbial community (Figure 2A). At the species level, we compared the alpha diversity of gut microbiota between the two groups at different time points using the Chao1 index and Simpson index for observed species counts. The results showed no significant differences in species richness or community diversity between SD and control mice (Figure 2B).

To investigate differences in species abundance and distribution between the two groups at each time point, we analyzed beta diversity of the gut microbiota separately for each week. Partial least squares discriminant analysis (PLS-DA) score plots demonstrated significant separation between SD and control mice at each time point, indicating marked differences in microbial community composition between the two groups (Figure 2C). We generated absolute abundance bar charts to display the species-level taxa that were differentially represented between the SD and control groups (Figure 2D). These results demonstrate that the gut microbial composition of SD mice differed significantly from that of controls.

During week 1, the abundances of g_*Odoribacter* and g_Lachnospiraceae_UCG-006 were significantly higher in SD mice than in controls, whereas g_*Lactobacillus* and g_Muribaculaceae_genus were more abundant in the control group.

During week 2, the gut microbiota of SD mice exhibited a shift characterized by increased relative abundance of opportunistic pathogens and facultative anaerobes. Compared with the control group, the SD group showed significantly elevated abundance of *Streptococcus*_genus (approximately 5.7-fold), a 2.6-fold increase in *Ligilactobacillus*_genus, and concomitant enrichment of the rare taxa *Berryella*_genus and unclassified genera within the family Gastranaerophilaceae. Notably, the genus Zag_111 was detected almost exclusively in the SD group, suggesting it may represent an early responder to sleep deprivation in this model. These microbial shifts suggest that 2 weeks of sleep deprivation were associated with changes in community composition favoring facultative anaerobes and opportunistic taxa.

By week 3, the pattern of gut dysbiosis shifted further–from the increased relative abundance of opportunistic pathogens observed in week 2 to a marked reduction in the relative abundance of multiple key commensal bacteria. *Lactobacillus*_genus abundance in the SD group was reduced approximately 17-fold, *Bifidobacterium*_genus approximately 10-fold, and *Dubosiella*_genus approximately 32-fold compared with controls. *Parasutterella*_genus was nearly absent in the SD group but consistently detected in the control group. These genera include recognized producers of SCFAs and taxa associated with intestinal barrier integrity in the literature; their concurrent reduction in relative abundance suggests a reduction in the potential functional capacity of the gut microecosystem, though this inference is based on relative-abundance changes rather than direct functional measurements. In contrast, *Odoribacter*_genus was significantly enriched in the SD group, and *Anaerotruncus*_genus abundance was also markedly elevated. The genus *Angelakisella* was detected exclusively in the SD group, further indicating that the gut microbial composition had undergone substantial remodeling after 3 weeks of sleep deprivation.

Taking all 3 weeks together, the abundances of g_*Alistipes*, g_*Odoribacter*, and g_Lachnospiraceae_UCG-006 were consistently higher in SD mice, whereas g_*Lactobacillus*, g_*Bifidobacterium*, and g_*Dubosiella* were more abundant in the control group. These findings demonstrate that SD significantly alters the gut microbial community.

From a temporal dynamics perspective (Figure 2E), individual weekly comparisons within the SD group revealed that the majority of bacterial genera reached peak abundance during week 2. For example, g_*Bifidobacterium*, g_*Litchfieldia*, and Prevotellaceae_genus surged in week 2 before declining sharply in week 3, potentially reflecting the loss of microbial stability accompanied by intestinal functional disruption and enhanced inflammatory responses. The initial transient rise followed by a subsequent decline in beneficial bacteria such as g_*Bifidobacterium* and g_*Ligilactobacillus* may reflect the exhaustion of compensatory stress-response mechanisms. A minority of genera exhibited sustained increases; for example, *Akkermansia* showed a progressive rise with a marked fold-increase in later weeks. Its persistent elevation may be associated with intestinal barrier damage, enhanced mucin metabolism, and energy metabolic stress, and may serve as a key microbial marker of the late-phase response to SD. Certain genera declined continuously over time; for instance, Lachnospiraceae_NK4A136_group exhibited a persistent decrease. Its dominance during the early phase of sleep deprivation followed by progressive decline likely reflects the suppression of beneficial microbial communities, potentially related to oxidative stress or inflammation induced by sleep deprivation.

We next sought to characterize the impact of sleep deprivation on microbial community structure. Analysis was conducted at the genus level rather than at other taxonomic levels to reduce analytical complexity. All identified bacterial genera across all samples were included in the analysis. To further identify specific taxa associated with SD, we performed linear discriminant analysis effect size (LEfSe) analysis with a linear discriminant analysis (LDA) score threshold of 5. LDA value histograms and cladograms for the different groups (Figures 2F,G) showed that in week 1, the dominant taxa in the SD group belonged to genera within the order Caldicoprobacterales; in week 2, *Candidatus_Arthromitus* was uniquely characteristic of the SD group; and in week 3, genera within the order Bifidobacteriales were the dominant taxa in the control group.

### 3.4 Hepatic metabolite profiling in SD mice

**FIGURE 3:**
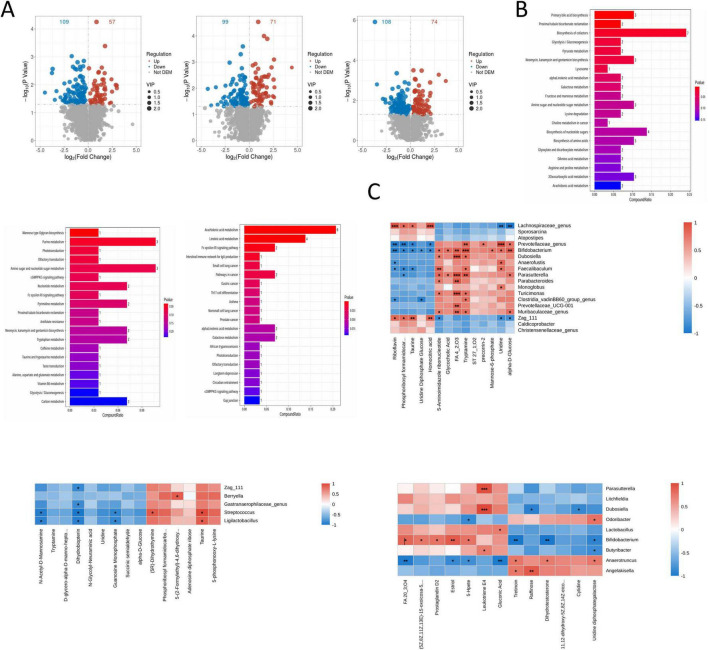
Hepatic metabolite profiling and microbiota–metabolite correlations in sleep-deprived mice. Panels follow a stepwise analytical flow–differential metabolites **(A)** → pathway-level interpretation **(B)** → integration with the gut microbiota **(C)**–and within each panel the three columns correspond to weeks 1, 2 and 3 (left to right) for visual consistency with Figure 2. **(A)** Volcano plots showing differentially expressed hepatic metabolites between CON and SD groups at weeks 1 (left panel: 57 up/109 down), 2 (middle panel: 71 up/99 down), and 3 (right panel: 74 up/108 down). Red dots represent upregulated metabolites, blue dots represent downregulated metabolites, and gray dots represent metabolites with no significant change; dot size indicates VIP score from PLS-DA. **(B)** Kyoto Encyclopedia of Genes and Genomes (KEGG) pathway enrichment analysis of differential hepatic metabolites at weeks 1, 2, and 3 (left-to-right). Compound ratio represents the proportion of differential metabolites enriched in each pathway, and color intensity represents statistical significance (*P*-value). **(C)** Correlation heatmaps showing Pearson correlation coefficients between differentially abundant gut bacterial genera and differential hepatic metabolites at weeks 1, 2, and 3. Red indicates positive correlation, blue indicates negative correlation; *p*-values were adjusted for multiple testing using the Benjamini–Hochberg procedure. *P < 0.05, **P < 0.01, ***P < 0.001. Data are presented as mean ± SD (*n* = 4 per group).

Metabolomic analysis was performed on liver tissue samples. In positive and negative ion modes, we detected 7046 peaks (negative mode, Supplementary Table 1) and 6034 peaks (positive mode, Supplementary Table 2), which may be induced by SD. After exclusion of natural isotope peaks, 997 and 1,902 metabolites were identified in positive and negative ion modes, respectively (Supplementary Table 3), encompassing amino acids, carbohydrates, bile acids, fatty acids, and vitamins. PLS-DA score plots revealed significant separation of hepatic metabolite profiles between the control and SD groups. Permutation tests confirmed the absence of overfitting and validated the PLS-DA models across all time periods. We further explored the effects of SD on hepatic metabolic alterations using volcano plots (Figure 3A) and Kyoto Encyclopedia of Genes and Genomes (KEGG) metabolic pathway enrichment analysis (Figure 3B).

#### 3.4.1 Week 1: early perturbation of bile acid metabolism and cofactor biosynthesis

In week 1, 57 metabolites were upregulated and 109 metabolites were downregulated in the SD group compared with controls. Subsequent annotation of differential metabolites using the KEGG database revealed enrichment in 48 metabolic pathways. Among these, primary bile acid biosynthesis was the most significantly enriched pathway (*P* < 0.05, containing 3 differential metabolites), indicating that sleep deprivation exerts a significant impact on bile acid metabolism at an early stage. Biosynthesis of cofactors exhibited the highest compound ratio (approximately 0.25, containing 7 differential metabolites), suggesting that sleep deprivation broadly affects the synthesis of B vitamins, folate, and other cofactors. Additionally, energy metabolism pathways such as glycolysis/gluconeogenesis and pyruvate metabolism were significantly enriched, indicating early perturbation of glucose metabolic homeostasis. Notably, arachidonic acid metabolism appeared among the enriched pathways in week 1 but with a relatively large *P*-value (*P* > 0.15), suggesting that inflammation-related lipid metabolism was not yet fully activated at this stage.

#### 3.4.2 Week 2: transitional phase involving nucleotide metabolism and tryptophan metabolism

In week 2, 71 metabolites were upregulated and 99 were downregulated in the SD group compared with controls, with differential metabolites enriched in 29 metabolic pathways. The composition of enriched pathways shifted markedly compared with week 1. Amino sugar and nucleotide sugar metabolism became the pathway with the highest compound ratio (approximately 0.10, containing 3 differential metabolites), followed closely by purine metabolism (compound ratio approximately 0.10, containing 3 differential metabolites), while mannose-type O-glycan biosynthesis was the most statistically significant pathway (*P* < 0.05). Furthermore, tryptophan metabolism and taurine and hypotaurine metabolism appeared among the enriched pathways, indicating that the metabolic conversion of tryptophan and taurine was significantly affected by the intermediate stage of sleep deprivation. Enrichment of pyrimidine metabolism and nucleotide metabolism indicated a deepening impact of sleep deprivation on nucleic acid metabolism by week 2. Notably, the primary bile acid biosynthesis pathway–the most significant pathway in week 1–was no longer enriched in week 2, and the arachidonic acid metabolism pathway had yet to emerge, indicating that week 2 represents a transitional phase between bile acid metabolic disruption and inflammatory metabolic activation.

#### 3.4.3 Week 3: full-scale activation of arachidonic acid metabolism and inflammatory immune pathways

In week 3, 74 metabolites were upregulated and 108 were downregulated in the SD group compared with controls, with differential metabolites enriched in 32 metabolic pathways. The pathway composition underwent a fundamental transformation compared with the previous 2 weeks. Arachidonic acid metabolism emerged as the most significant and metabolite-rich pathway (*P* < 0.025, compound ratio approximately 0.21, containing 6 differential metabolites), in stark contrast to its appearance with a large *P*-value in week 1. Linoleic acid metabolism ranked second (*P* < 0.025, containing 4 differential metabolites). Both pathways belong to polyunsaturated fatty acid metabolism, suggesting markedly enhanced lipid peroxidation and inflammatory mediator generation in the late phase of SD. More strikingly, a large number of inflammation- and immune-related pathways emerged among the enriched pathways in week 3, including the Fc epsilon RI signaling pathway (containing 2 differential metabolites, *P* < 0.025), Th17 cell differentiation, intestinal immune network for IgA production, and pathways in cancer. None of these pathways were present in weeks 1 or 2, indicating a progression from metabolic disruption to systemic inflammatory immune activation in the late phase of sleep deprivation. Additionally, alpha-linolenic acid metabolism and galactose metabolism were significantly enriched, further confirming multi-pathway perturbation of lipid and carbohydrate metabolism.

#### 3.4.4 Temporal evolution of metabolic pathways

Integrating the KEGG pathway enrichment analyses across all 3 weeks, sleep deprivation-induced hepatic metabolic disturbances exhibited a clear temporal evolutionary pattern: week 1 was dominated by perturbations in primary bile acid biosynthesis and cofactor synthesis, representing the early breakdown of metabolic homeostasis; week 2 shifted toward amino sugar and nucleotide sugar metabolism, purine metabolism, and tryptophan metabolism, reflecting widespread remodeling of metabolic networks; week 3 featured full-scale activation of arachidonic acid and linoleic acid metabolism accompanied by large-scale enrichment of inflammatory immune pathways, marking the transition from metabolic disruption to inflammatory injury. This pathway evolution trajectory–from “bile acid disruption” to “metabolic remodeling” to “inflammatory cascade”–was highly consistent with the results of microbiota-metabolite correlation analyses.

### 3.5 Correlation analysis between SD-associated gut microbiota and hepatic metabolites

To further explore the relationship between SD-associated gut microbial taxa and hepatic metabolites, we performed correlation analysis between differentially abundant bacterial genera and differential hepatic metabolites at each time point, as shown in Figure 3C. The results revealed a pronounced temporal signature in the microbiota-metabolite correlation network: from the bile acid and protective metabolite axis in week 1, to microbiota-metabolite homeostatic decoupling in week 2, and inflammatory metabolic pathway activation in week 3–collectively describing a temporal pattern of SD-associated metabolic alterations that co-vary with gut microbiota changes.

#### 3.5.1 Week 1: decline of beneficial bacteria and coordinated reduction of protective metabolites

In week 1, the correlation analysis revealed the most extensive and dense network of microbiota-metabolite associations. LEfSe analysis showed that *Bifidobacterium*_genus (LDA score approximately 6.5), Prevotellaceae_genus, and *Faecalibaculum*_genus were significantly enriched in the control group–indicating significantly reduced abundance in the SD group; whereas *Sporosarcina*_genus, Lachnospiraceae_genus, *Atopostipes*_genus, *Zag_111*_genus, and Christensenellaceae_genus were enriched in the SD group.

Several bacterial genera enriched in the SD group showed strong positive correlations with metabolites that were elevated in the SD group. Specifically, Lachnospiraceae_genus was significantly and positively correlated with taurine (*P* < 0.05), homocitric acid (*P* < 0.05), and riboflavin (*P* < 0.05). Similarly, *Sporosarcina*_genus and *Atopostipes*_genus were positively correlated with taurine and homocitric acid, suggesting that these bacteria may promote the accumulation of these metabolites under conditions of sleep deprivation.

Conversely, bacterial genera that were reduced in the SD group–particularly *Dubosiella*_genus, *Bifidobacterium*_genus, and *Faecalibaculum*_genus–showed significant positive correlations with tryptamine (*P* < 0.05), glycocholic acid, and alpha-D-glucose, and negative correlations with taurine and homocitric acid. Notably, tryptamine–a product of microbial decarboxylation of tryptophan–exhibited significant correlations with multiple bacterial genera. Tryptamine generated through gut microbial metabolism of tryptophan has been demonstrated in murine studies to activate the AhR pathway, suppress hepatic inflammation, and protect the intestinal barrier (Lamas et al., 2016). The microbial synthesis of tryptamine primarily depends on specific bacterial taxa expressing tryptophan decarboxylase, including *Clostridium sporogenes* and *Ruminococcus gnavus* (Williams et al., 2014). The strong positive correlation between *Bifidobacterium* and tryptamine is noteworthy; although *Bifidobacterium* is not a direct producer of tryptamine, bifidobacteria can modulate tryptophan metabolism (Wang et al., 2025). The synchronous reduction of both beneficial bacteria and tryptamine suggests that sleep deprivation may impair microbial tryptophan metabolic pathways, potentially exacerbating neuropsychiatric and gastrointestinal symptoms associated with sleep loss.

The reductions in uridine and 5-aminoimidazole ribonucleotide, along with decreases in alpha-D-glucose and mannose-6-phosphate, indicate broader disruption of energy metabolism and nucleotide biosynthesis pathways. Moreover, the significant positive correlation between glycocholic acid–a primary conjugated bile acid–and *Bifidobacterium* is consistent with the KEGG enrichment analysis identifying “primary bile acid biosynthesis” as the most significantly enriched pathway in week 1, suggesting that the decline of beneficial bacteria may disrupt normal bile acid metabolic cycling in the early stages of sleep deprivation. Therefore, the coordinated decline in beneficial bacterial abundance during early sleep deprivation may weaken the host’s early defense against liver injury by reducing the production of protective metabolites such as tryptamine and disrupting bile acid metabolism.

#### 3.5.2 Week 2: metabolic homeostatic decoupling and probiotic–pathogen metabolic niche competition during the microbial turbulence phase

Compared with week 1, the number of significant microbiota-metabolite associations decreased sharply in week 2, with only five differentially abundant genera entering the correlation analysis. LEfSe analysis showed that the bacterial genera enriched in the SD group during this period included *Streptococcus*, *Berryella*, *Zag_111*, Gastranaerophilaceae_genus, and *Ligilactobacillus*. *Streptococcus* and *Ligilactobacillus* exhibited similar metabolite association patterns: both showed significant negative correlations with dihydrobiopterin (*P* < 0.05), N-glycolylneuraminic acid (*P* < 0.05), and guanosine monophosphate (*P* < 0.05), and significant positive correlations with taurine (*P* < 0.05). Despite their parallel correlation patterns and co-enrichment in the SD group, the biological significance of these two taxa is fundamentally different. *Ligilactobacillus*, as a probiotic member of the Lactobacillaceae family, may have undergone compensatory expansion following the substantial loss of core probiotics such as *Bifidobacterium* and *Dubosiella* in week 1–representing an attempted restorative response by the gut microecosystem. However, the simultaneous enrichment of *Streptococcus* as an opportunistic pathogen may have offset this compensatory effect. The parallel associations of both taxa with identical metabolites suggest that they may occupy the same metabolic niche, engaging in direct competition between probiotics and pathogens amid the microbial turbulence of the intermediate phase of sleep deprivation. This finding echoes the tryptophan metabolism and taurine and hypotaurine metabolism pathways identified in the KEGG enrichment analysis for week 2, suggesting that competitive microbial restructuring may modulate the hepatic metabolite profile through effects on tryptophan and taurine metabolic conversion. Furthermore, the overall minimal number of significant microbiota-metabolite associations in week 2 is consistent with the observation that microbial abundance reached its fluctuation peak during this period, suggesting that the homeostatic regulatory network between microbiota and metabolites underwent a transient decoupling at this stage–the microbial community was in a phase of rapid restructuring and had not yet established new, stable metabolic associations.

#### 3.5.3 Week 3: activation of inflammatory metabolic pathways and differential regulation by beneficial bacteria

The week 3 correlation analysis was dominated by inflammatory metabolites. The differential metabolites were predominantly products of the arachidonic acid metabolic pathway, including prostaglandin D2 (PGD2), 5-HPETE, leukotriene E4 (LTE4), and 11,12-dihydroxy-5Z,8Z,14Z-eicosatrienoic acid–highly consistent with the KEGG enrichment analysis showing arachidonic acid metabolism as the top-ranked pathway (containing 6 differential metabolites, *P* < 0.025). LEfSe analysis revealed that *Dubosiella*, *Litchfieldia*, *Lactobacillus*, *Butyribacter*, and *Parasutterella* were enriched in the control group, whereas *Anaerotruncus*, *Angelakisella*, and *Odoribacter* were enriched in the SD group. *Lactobacillus* abundance was significantly and positively correlated with PGD2, LTE4, and 5-HPETE levels (all decreased in the SD group), and significantly and negatively correlated with dihydrotestosterone (DHT) and tretinoin levels. Similarly, *Bifidobacterium* abundance was positively correlated with multiple oxidized lipid mediators and negatively correlated with retinoic acid. The reduction of these bacteria may be associated with attenuated suppression of the arachidonic acid metabolic pathway, potentially permitting the accumulation of pro-inflammatory leukotrienes and eicosanoids, although this hypothesis requires direct functional validation. At week 3, the gut microbiota exhibited a qualitative shift, with key commensal genera (*Lactobacillus*, *Bifidobacterium*, *Dubosiella*) showing concurrent, marked reductions in relative abundance. Conversely, *Odoribacter*, which was enriched in the SD group, showed positive correlations with tretinoin and DHT levels, and negative correlations with PGD2 and LTE4, suggesting a possible association with late-phase hepatic metabolic alterations that would require experimental confirmation.

## 4 Discussion

### 4.1 Week 1–early dismantling of the protective metabolic barrier

The central finding of week 1 was the reduction of beneficial bacteria accompanied by a coordinated decline in protective metabolites. *Bifidobacterium*, *Dubosiella*, and *Faecalibaculum*–all recognized SCFA-producing bacteria (Qu et al., 2018; Ai et al., 2021; Miao et al., 2022)–were significantly depleted in the SD group, concurrent with coordinated reductions in tryptamine, glycocholic acid, and alpha-D-glucose. This finding is consistent with previous reports that sleep deprivation can alter gut microbial composition at an early stage (Poroyko et al., 2016).

Tryptamine, produced via microbial tryptophan decarboxylase–catalyzed decarboxylation of tryptophan, has been implicated in gut–liver axis signaling. Prior studies report that tryptamine can contribute to intestinal barrier protection and suppression of hepatic inflammation through activation of the AhR signaling pathway (Zelante et al., 2013). The positive correlation between tryptamine and both *Bifidobacterium* and *Dubosiella* observed in our study, together with their concurrent decline in the SD group, is consistent with a possible attenuation of AhR-ligand availability following SD-associated changes in microbial tryptophan metabolism. However, because this inference is based on correlation only, and because AhR pathway activity, receptor occupancy, and downstream effectors were not directly measured, this interpretation should be considered hypothesis-generating and requires validation by protein-level assays (for example, CYP1A1 induction) and functional AhR reporter experiments. Notably, although *Bifidobacterium* is not a direct producer of tryptamine, bifidobacteria can indirectly promote tryptophan metabolism by modulating intestinal pH and providing metabolic precursors such as indolelactic acid (Laursen et al., 2021); their loss may therefore affect tryptamine generation through cascading effects within the inter-microbial metabolic network.

Concurrently, the positive correlation between glycocholic acid and *Bifidobacterium*, together with their decreased levels in the SD group, corroborates the KEGG enrichment analysis result identifying primary bile acid biosynthesis as the most significantly enriched pathway in week 1. Bile acids regulate hepatic lipid metabolism and gluconeogenesis through the FXR and TGR5 signaling axes (Wahlström et al., 2016). Intestinal bacteria are known to regulate bile acid deconjugation and reabsorption through bile salt hydrolase activity (Ridlon et al., 2014). Based on these literature relationships, the reduced relative abundance of *Bifidobacterium* and other bile-acid–modifying taxa in week 1 could plausibly alter the bile salt hydrolase activity pool and, in turn, downstream FXR/TGR5-mediated signaling, although direct evidence for altered FXR/TGR5 signaling was not obtained in the present study and will require dedicated protein-level and reporter-based investigation.

Additionally, the elevation of taurine in the SD group and its positive correlation with SD-enriched taxa such as *Sporosarcina* and Lachnospiraceae merit attention. In the liver, taurine primarily participates in bile acid conjugation reactions in the form of taurocholic acid. Its abnormal accumulation may reflect decreased efficiency of bile acid conjugation metabolism–when primary bile acid biosynthesis is impaired, free taurine, as a conjugation substrate, may accumulate in the liver due to reduced utilization. This finding is descriptively consistent with the KEGG analysis result showing impairment of the primary bile acid biosynthesis pathway in week 1. Taurine itself possesses antioxidant and osmotic regulatory functions, and its early-stage elevation may also carry compensatory protective significance (Schaffer and Kim, 2018); however, such compensation may not be sustainable in subsequent stages.

### 4.2 Week 2–ecological niche competition and transitional metabolic network remodeling

The microbiota-metabolite correlation analysis in week 2 revealed a distinctive “metabolic homeostatic decoupling” phenomenon: microbial abundance reached its fluctuation peak, yet the number of significant microbiota-metabolite associations paradoxically fell to its nadir across the 3-weeks period. This seemingly contradictory observation can be explained from a microbial ecology perspective.

The concurrent enrichment of *Streptococcus* (approximately 5.7-fold increase) and *Ligilactobacillus* (approximately 2.6-fold increase) in the SD group at week 2 constitutes a particularly striking finding. Both taxa exhibited highly similar metabolite association patterns–negative correlations with dihydrobiopterin, N-glycolylneuraminic acid, and guanosine monophosphate, and positive correlations with taurine–yet their biological attributes are fundamentally different. The increased relative abundance of *Ligilactobacillus*, a member of the Lactobacillaceae family, following the earlier decrease in core commensals (*Bifidobacterium*, *Dubosiella*) in week 1 may represent a compensatory response by the gut microecosystem. The concurrent increase in *Streptococcus*, a facultative anaerobic taxon that includes opportunistic pathogens, raises the possibility that the ecological space vacated by commensal loss was partially occupied by less beneficial taxa. The observed parallelism in metabolite correlations for these two genera is compatible with, but does not by itself demonstrate, shared metabolic niche occupancy; direct competition studies (e.g., co-culture or gnotobiotic colonization experiments) would be required to establish this. The pattern is nonetheless consistent with the colonization resistance framework described by Buffie et al. (2015), in which functionally similar but biologically distinct taxa may occupy vacated nutritional and metabolic sites.

Dihydrobiopterin, the oxidized form of tetrahydrobiopterin (BH4), serves as an important cofactor for nitric oxide synthase (NOS). A decrease in the BH4/BH2 ratio leads to NOS uncoupling, shifting its activity from nitric oxide production to superoxide anion generation, thereby exacerbating oxidative stress (Crabtree and Channon, 2011). The pattern of decreased dihydrobiopterin and elevated taurine in the SD group may reflect a complex compensatory response in the liver under redox imbalance. N-glycolylneuraminic acid, a member of the sialic acid family that plays an important role in glycoprotein and glycolipid modification, showed changes consistent with the significant enrichment of amino sugar and nucleotide sugar metabolism and mannose-type O-glycan biosynthesis pathways identified in the KEGG analysis for week 2, suggesting that glycosylation modification pathways were already significantly affected at this stage.

Notably, the appearance of tryptophan metabolism and taurine metabolism pathways in the week 2 KEGG analysis temporally resonated with the positive correlations of *Streptococcus* and *Ligilactobacillus* with taurine observed in the microbiota correlation analysis. This suggests that week 2, as a transitional period from bile acid metabolic disruption (week 1) to inflammatory metabolic activation (week 3), is characterized by rapid microbial restructuring and transient decoupling of the metabolic network. The microbiota was still in the process of “selecting a new homeostatic configuration,” with temporarily diminished regulatory efficacy over metabolites, thus accumulating conditions for the full-scale activation of inflammatory pathways in week 3.

### 4.3 Week 3–inflammatory metabolic cascade driven by probiotic collapse

The results from week 3 represent the terminal stage of sleep deprivation-induced gut-liver axis disruption, characterized by two prominent features: firstly, the synchronous large-scale collapse of key probiotic genera; secondly, the full-scale activation of arachidonic acid metabolism and inflammatory immune pathways.

The synchronous depletion of *Lactobacillus* (reduced 17-fold), *Bifidobacterium* (reduced 10-fold), and *Dubosiella* (reduced 32-fold) in the SD group represents a pronounced concurrent decrease in the relative abundance of three core commensal genera. All three are recognized SCFA producers, contributing to pools of butyrate and acetate. SCFAs have been reported to support tight junction protein expression and intestinal barrier integrity through activation of intestinal epithelial G protein-coupled receptors (GPR43/GPR109A) and to exert anti-inflammatory effects in the liver in part via modulation of the NF-κB signaling pathway (Smith et al., 2013; Canfora et al., 2015). Their concurrent reduction in relative abundance implies not only a probable decrease in SCFA-producing capacity but also, given the parallel increase in serum LPS confirmed by ELISA in our study, a context compatible with increased intestinal permeability. In the literature, increased intestinal permeability is reported to permit translocation of microbe-associated molecular patterns, including LPS, which can activate the TLR4/NF-κB signaling pathway in Kupffer cells and contribute to inflammatory cascades (Tripathi et al., 2018). We did not directly measure intestinal permeability, TLR4 activation, or downstream NF-κB signaling in the present study, so engagement of this specific axis in our model remains inferential.

The arachidonic acid metabolism pathway emerged as the most significantly enriched pathway in week 3 (containing 6 differential metabolites, *P* < 0.025), in stark temporal contrast to its appearance with only a large *P*-value in week 1. Oxidized lipid mediators including PGD2, LTE4, 5-HPETE, and 15-oxo-ETE showed significant differences between the SD and control groups. PGD2, a major product of cyclooxygenase-2, plays dual roles in inflammation and immune regulation (Ricciotti and FitzGerald, 2011). LTE4, the terminal product of the cysteinyl leukotriene pathway, is a potent pro-inflammatory lipid mediator involved in increased vascular permeability and leukocyte recruitment (Singh et al., 2010). 5-HPETE, the direct product of 5-lipoxygenase, serves as a key precursor for leukotriene synthesis. The significant alterations of these arachidonic acid metabolites signify the full-scale activation of hepatic lipid peroxidation and inflammatory mediator generation pathways.

The positive correlations of *Lactobacillus* with PGD2, LTE4, and 5-HPETE are consistent with a relationship between this taxon and inflammatory lipid metabolism, though they do not by themselves establish a direct causal link. Prior *in vitro* and *in vivo* reports indicate that lactobacilli can attenuate cyclooxygenase-2 and 5-lipoxygenase expression through the production of conjugated linoleic acid and SCFAs, thereby influencing the arachidonic acid cascade (Iwakiri et al., 2002; Kishino et al., 2013). Their large-scale depletion implies the release of this inhibitory regulation, permitting the full activation of arachidonic acid metabolic pathways under inflammatory stimuli. Additionally, the positive correlations of *Bifidobacterium* with multiple oxidized lipid mediators further support the hypothesis that probiotics exert remote regulatory effects on hepatic lipid signaling through SCFAs and other metabolites (Schroeder and Bäckhed, 2016).

Of particular note, *Odoribacter*, which was enriched in the SD group, showed positive correlations with tretinoin (retinoic acid) and DHT, and negative correlations with PGD2 and LTE4. *Odoribacter* belongs to the phylum Bacteroidetes, and certain species produce succinate rather than butyrate as their primary fermentation end product (Göker et al., 2011). Its increased relative abundance following the earlier reduction in commensals raises the possibility that it may influence hepatic metabolism through alteration of the intestinal organic acid profile (succinate replacing butyrate), although this remains speculative without direct measurement of portal-vein metabolites. The significant elevation of tretinoin (all-trans retinoic acid) in the SD group (approximately 2.4-fold) is a notable observation. The liver is the primary organ for retinol (vitamin A) storage; under normal conditions, hepatic stellate cells store retinol in lipid droplets, and under inflammatory or fibrotic stimuli, stellate cells can become activated and release and convert substantial amounts of retinol to retinoic acid (Blaner et al., 2016). Elevated retinoic acid may therefore warrant follow-up evaluation of stellate cell activation in SD models, though direct markers of stellate cell activation (e.g., α-SMA, desmin) were not assessed here. The elevation of DHT may reflect changes in hepatic steroid metabolism under chronic stress, potentially related to hypothalamic–pituitary–adrenal axis activity. The positive correlations of *Odoribacter* with these metabolic changes are compatible with a possible contributory role for this genus but do not establish causation; dedicated colonization or antibiotic-depletion experiments would be required to test this.

Furthermore, the first-time appearance of inflammatory immune pathways including the Fc epsilon RI signaling pathway, Th17 cell differentiation, and intestinal immune network for IgA production in week 3 indicates that metabolic disruption has escalated from mere changes in metabolite levels to systemic immune-inflammatory responses. The Fc epsilon RI signaling pathway is directly linked to mast cell degranulation and the release of arachidonic acid metabolites (Galli et al., 2008), while Th17 cell differentiation has been associated with intestinal barrier disruption and microbial translocation (Ivanov et al., 2009). The concurrent enrichment of these immune-related pathways in week 3 is consistent with the possibility that later-stage hepatic metabolic alterations may involve contributions from multiple processes–including microbial-product translocation, lipid inflammatory mediators, and adaptive immune responses–but we emphasize that pathway enrichment from metabolomics data reflects metabolite-level signatures rather than direct activation of the corresponding immune pathways, which would need to be verified by protein- and cell-level assays.

### 4.4 Three-stage descriptive framework for the temporal pattern of the gut–liver axis

Integrating the multi-omics correlation analyses across all three weekly time points, we propose a “three-stage descriptive framework” for the temporal pattern of SD-associated alterations along the gut–liver axis. We emphasize that the boundaries between stages were not defined by pre-specified statistical criteria; rather, they were delineated *post hoc* from the dominant microbiota, metabolite, and pathway features observed at each weekly time point. The framework is therefore intended as a descriptive summary of the observed temporal pattern–not as a mechanistically validated model of SD-induced liver injury–and the transitions between stages should be understood as qualitative rather than quantitative:

Stage 1 (Week 1)– “Early perturbation phase”: Characterized by reduced relative abundance of core commensal genera such as *Bifidobacterium* and *Dubosiella*, accompanied by decreased levels of potential AhR ligands including tryptamine and by perturbation of primary bile acid metabolism. No prominent inflammatory pathway enrichment was observed at this stage. The mild elevations in ALT/AST during this stage are consistent with subclinical biochemical changes rather than overt hepatic injury.

Stage 2 (Week 2)– “Transitional remodeling phase”: Characterized by concurrent increased relative abundance of *Ligilactobacillus* (a Lactobacillaceae member) and *Streptococcus* (a facultative anaerobe that includes opportunistic taxa), a lower density of significant microbiota–metabolite correlations, and metabolic pathway remodeling involving nucleotide sugar, tryptophan, and taurine metabolism. ALT/AST reached their peak values at this time point, coinciding with, but not demonstrated to be caused by, the observed microbial and metabolic changes.

Stage 3 (Week 3)– “Inflammatory metabolic signature phase”: Characterized by marked concurrent reductions in the relative abundance of *Lactobacillus*, *Bifidobacterium*, and *Dubosiella*, together with enrichment of arachidonic acid metabolic pathways and several inflammatory immune pathways at the metabolomic level, alongside increased serum LPS. The increased relative abundance of *Odoribacter* at this time point may contribute to the observed metabolic changes, though this remains to be demonstrated experimentally.

The temporal dynamics of ALT/AST–peaking at week 2 and declining slightly at week 3 while remaining above baseline–warrant in-depth discussion within this model. The week 2 ALT/AST peak may reflect the acute stress injury sustained by the liver during the transition from loss of protective microbial function in week 1 to pathogen expansion in week 2–when compensatory microbial repair was not yet in place and new pathogenic signals were already surging. By week 3, although inflammatory pathways were fully activated, the liver may have generated a partial adaptive response through induction of antioxidant enzymes and acute phase proteins. However, the sustained activation of arachidonic acid metabolic pathways and immune pathways indicates that this “apparent adaptation” actually masks deeper metabolic-immune disruption, which over the long term may lead to chronic liver injury and progression of fibrosis. This parallels the clinical phenomenon in which transaminase levels in patients with chronic liver disease may normalize despite ongoing progression of fibrosis (Kim et al., 2008).

### 4.5 Sustained rise of *Akkermansia*: a potential but debated marker

From the perspective of longitudinal microbial dynamics, *Akkermansia* exhibited a progressive increase in the SD group, with a marked fold-increase in later weeks. *Akkermansia muciniphila* is a mucin-degrading bacterium whose abundance has been related to the status of the intestinal mucus layer, although its role in host health remains a subject of ongoing debate in the literature. Under some conditions, moderate levels of *Akkermansia* have been associated with beneficial metabolic outcomes (Everard et al., 2013); in other settings, disproportionate proliferation has been linked to excessive mucin degradation and compromised barrier integrity (Ganesh et al., 2013). In our SD model, the progressive rise of *Akkermansia* coincided temporally with the elevation in serum LPS at week 3, which is compatible with–though it does not establish–a link to altered intestinal barrier function. Given the divergent reports in the literature, the rise of *Akkermansia* observed here should be interpreted cautiously as a candidate marker rather than a definitive indicator of intestinal barrier damage, and future studies employing direct barrier-function readouts will be required to clarify its role in the context of sleep deprivation.

### 4.6 Hypothesis-generating directions for future research

Several observations in this exploratory study suggest directions for future preclinical research, although we emphasize that this study is limited to a mouse model and several unaddressed confounders (see Section “4.7 Study limitations”). Clinical recommendations cannot be drawn from the present data, and the following points are presented solely as hypotheses for subsequent testing. First, because the most pronounced changes in microbial relative abundance and metabolic pathway enrichment emerged between weeks 2 and 3, future studies could examine whether interventions introduced before this transition–for example, defined probiotic supplementation (such as *Bifidobacterium* or *Lactobacillus* strains), exogenous supplementation of candidate AhR ligands (such as tryptamine), or SCFA precursors (such as sodium butyrate)–modify the observed temporal trajectory in SD models. Such preclinical studies, together with mechanistic and causal validation, would be required before any potentially modifiable time period observed in this descriptive mouse-model dataset could be regarded as relevant to clinical intervention timing. Second, the elevation of retinoic acid at week 3 may warrant follow-up investigation as a candidate marker of stellate cell activation in SD models, contingent on direct assessment of stellate cell activation markers and histological indices of fibrosis, which were not evaluated here. Third, *Odoribacter*, which increased in relative abundance in the SD group and correlated with several late-phase metabolites, could be examined in future work as a candidate taxon for mechanistic studies, but its utility as any form of biomarker cannot be inferred from the present correlational data alone.

The gut–liver axis has been proposed as an intermediary pathway relevant to sleep deprivation and liver-related outcomes. Benedict et al. reported in 2016 that short-term SD in humans can alter the Firmicutes-to-Bacteroidetes ratio in the gut microbiome (Benedict et al., 2016); our mouse-model data extend such observations by describing concurrent hepatic metabolic changes over a 3-weeks timeframe. Whether these descriptive observations will ultimately support multi-step intervention strategies in humans remains a hypothesis that future mechanistic and interventional studies–including in non-SD dysbiosis models and, eventually, in humans–will need to test.

### 4.7 Study limitations

Several important limitations of this study should be noted. First, the platform-based protocol used here is a modified version of the multiple-platform-over-water paradigm, which has been validated by prior electroencephalography-based studies to specifically suppress >90% of rapid eye movement sleep (Suchecki et al., 2000; Machado et al., 2004), and behavioral monitoring of activity patterns by overhead night-vision infrared cameras across the experiment provided in-cage evidence consistent with effective sleep deprivation in the SD group. It should also be noted that the night-vision video monitoring described in Section “3.1 SD altered body weight, liver weight, and liver-to-body weight ratio in mice” was used as a qualitative complementary observation only; quantitative behavioral metrics (e.g., mean rest duration per session or postural-adjustment frequency) were not extracted from the recordings, so the in-cage video evidence cannot by itself substitute for objective sleep-staging or actigraphic quantification. Even so, electroencephalography, actigraphy, and serum corticosterone were not directly recorded in the present cohort, so the precise within-animal degree and pattern of rapid eye movement sleep/non-rapid eye movement sleep loss cannot be independently quantified here; future work incorporating these objective sleep-physiology readouts will allow more precise characterization of the sleep-deprivation phenotype in this model. Second, the platform-based SD model inherently introduces physical restraint, chronic psychological stress, and altered feeding behavior as unavoidable confounders. To address this, in the present study the control group was housed on a 5 cm diameter cylindrical large-platform placed in identical water tanks under matched handling and circadian conditions, thereby controlling for physical restraint, water-tank exposure, social-housing arrangement, and timing while permitting normal sleep on the larger platform. This large-platform comparator is the field’s standard control for the multiple-platform-over-water paradigm and has been used precisely because it equalizes the non-specific water-tank, restraint, and isolation stressors between groups while permitting normal sleep on the larger platform; the systematic difference between the two arms is therefore expected to be sleep loss *per se* rather than generic environmental stress. As an additional coarse readout of overall stress and feeding-state equivalence, body weight was tracked in all animals throughout the 3-weeks experiment; the modest weight loss observed in the SD group is consistent with a sleep-loss-specific metabolic perturbation rather than a gross feeding or stress-state imbalance between groups. Even so, full equivalence of stress exposure between the SD and large-platform-control groups cannot be guaranteed without dedicated readouts (e.g., serum corticosterone), and subtle differences in mobility, feeding patterns, or anxiety-related behavior may persist; objective stress-physiology characterization will be needed in future work to fully isolate sleep loss from residual platform-related stress. As a result, this residual concern is partially mitigated by the large-platform control but should still be considered when interpreting our findings, and complete stress-physiology validation remains an important objective for follow-up studies. Third, the sample size was four mice per group per weekly time point, which, although consistent with common practice for multi-omics pilot studies, limits statistical power and increases the risk of false-positive findings in high-dimensional analyses; Only male C57BL/6J mice were used; given well-documented sexual dimorphism in sleep regulation and metabolic responses, replication in female mice is necessary. Fourth, the study relies on microbiota–metabolite correlation analyses and does not establish causal relationships; causal validation through fecal microbiota transplantation, germ-free colonization, gnotobiotic mono-colonization, defined strain supplementation, or targeted metabolite administration was not performed. Fifth, the mechanistic pathways discussed in this manuscript (AhR, FXR/TGR5, TLR4/NF-κB) were inferred from metabolite-level and microbiome-level signatures together with literature-based relationships; no direct protein-level measurements were performed, so engagement of these specific signaling axes in our model remains inferential. Sixth, only the colonic microbiota was examined, without assessment of small-intestinal segments that may play more direct roles in bile acid metabolism and nutrient absorption. Seventh, key microbial metabolites such as SCFAs were not directly quantified; their mediating role in gut–liver axis signaling awaits confirmation through targeted metabolomics. Finally, intestinal barrier function was inferred from serum LPS alone; direct readouts such as tight-junction protein quantification were not performed.

## 5 Conclusion

In summary, integrating the KEGG pathway enrichment analyses and microbiota–metabolite correlation analyses, SD-associated alterations in the gut microbiota and hepatic metabolome exhibited a broadly parallel temporal pattern of co-occurring changes in this mouse model: week 1 was characterized by reduced relative abundance of commensal bacteria, coordinated decline of candidate protective metabolites, and perturbation of the primary bile acid biosynthesis pathway; week 2 featured a reduction in microbiota–metabolite correlation density, concurrent enrichment of a compensatory Lactobacillaceae member and an opportunistic taxon, and metabolic pathway remodeling toward nucleotide sugar and tryptophan metabolism; week 3 was marked by enrichment of arachidonic acid metabolism and inflammatory immune pathways, alongside concurrent reductions in the relative abundance of multiple commensal genera and accumulation of candidate pro-inflammatory metabolites. This temporally ordered pattern–described here as a “protective loss–homeostatic decoupling–inflammatory signature” descriptive framework–offers a hypothesis-generating perspective on how SD-associated gut microbial changes may parallel hepatic metabolic alterations. Given the correlational nature of the analyses, the results should be regarded as preliminary and do not support clinical recommendations; causal validation will be required before any translational implications can be drawn.

## Data Availability

The 16S rRNA gene sequencing data presented in this study are deposited in the NCBI Sequence Read Archive (SRA) repository under BioProject accession number PRJNA1467287 (https://www.ncbi.nlm.nih.gov/bioproject/PRJNA1467287). The untargeted LC-MS/MS metabolomics data are deposited in the MetaboLights repository, accession number MTBLS14541 (https://www.ebi.ac.uk/metabolights/MTBLS14541). Further inquiries can be directed to the corresponding author.

## Supplementary material

Supplementary Table 1 Negative ion peaks.

Supplementary Table 2 Positive ion peaks.

Supplementary Table 3 Number of metabolites identified in positive and negative ion modes.

## References

[B1] Aho V. Ollila H. M. Kronholm E. Soininen P. Kangas A. J. Hilvo M. et al. (2016). Prolonged sleep restriction induces changes in pathways involved in cholesterol metabolism and inflammatory responses. *Sci. Rep.* 6:24828. 10.1038/srep24828 27102866 PMC4840329

[B2] Ai X. Wu C. Yin T. Zhur O. Liu C. Yan X. et al. (2021). Antidiabetic function of *Lactobacillus fermentum* MF423-fermented rice bran and its effect on gut microbiota structure in type 2 diabetic mice. *Front. Microbiol.* 12:682290. 10.3389/fmicb.2021.682290 34248898 PMC8266379

[B3] Albillos A. de Gottardi A. Rescigno M. (2020). The gut-liver axis in liver disease: pathophysiological basis for therapy. *J. Hepatol.* 72 558–577. 10.1016/j.jhep.2019.10.003 31622696

[B4] Benedict C. Vogel H. Jonas W. Woting A. Blaut M. Cedernaes J. et al. (2016). Gut microbiota and glucometabolic alterations in response to recurrent partial sleep deprivation in normal-weight young individuals. *Mol. Metab.* 5 1175–1186. 10.1016/j.molmet.2016.10.003 27900260 PMC5123208

[B5] Blaner W. S. Li Y. Brun P. J. Yuen J. J. Lee S. A. Clugston R. D. (2016). Vitamin A absorption, storage and mobilization. *Subcell Biochem.* 81 95–125. 10.1007/978-94-024-0945-1_4 27830502

[B6] Bolyen E. Rideout J. R. Dillon M. R. Bokulich N. A. Abnet C. C. Al-Ghalith G. A. et al. (2019). Reproducible, interactive, scalable and extensible microbiome data science using QIIME 2. *Nat. Biotechnol.* 37 852–857. 10.1038/s41587-019-0209-9 31341288 PMC7015180

[B7] Buffie C. G. Bucci V. Stein R. R. Ling L. Gobourne A. No D. et al. (2015). Precision microbiome reconstitution restores bile acid mediated resistance to *Clostridium difficile*. *Nature* 517 205–208. 10.1038/nature13828 25337874 PMC4354891

[B8] Callahan B. J. McMurdie P. J. Rosen M. J. Han A. W. Johnson A. J. Holmes S. P. (2016). DADA2: high-resolution sample inference from Illumina amplicon data. *Nat. Methods* 13 581–583. 10.1038/nmeth.3869 27214047 PMC4927377

[B9] Canfora E. E. Jocken J. W. Blaak E. E. (2015). Short-chain fatty acids in control of body weight and insulin sensitivity. *Nat. Rev. Endocrinol.* 11 577–591. 10.1038/nrendo.2015.128 26260141

[B10] Canfora E. E. Meex R. C. R. Venema K. Blaak E. E. (2019). Gut microbial metabolites in obesity, NAFLD and T2DM. *Nat. Rev. Endocrinol.* 15 261–273. 10.1038/s41574-019-0156-z 30670819

[B11] Chattu V. K. Manzar M. D. Kumary S. Burman D. Spence D. W. Pandi-Perumal S. R. (2018). The global problem of insufficient sleep and its serious public health implications. *Healthcare* 7:1. 10.3390/healthcare7010001 30577441 PMC6473877

[B12] Crabtree M. J. Channon K. M. (2011). Synthesis and recycling of tetrahydrobiopterin in endothelial function and vascular disease. *Nitric Oxide* 25 81–88. 10.1016/j.niox.2011.04.004 21550412 PMC5357050

[B13] Đukanović N. La Spada F. Emmenegger Y. Niederhäuser G. Preitner F. Franken P. (2022). Depriving mice of sleep also deprives of food. *Clocks Sleep* 4 37–51. 10.3390/clockssleep4010006 35225952 PMC8884003

[B14] Everard A. Belzer C. Geurts L. Ouwerkerk J. P. Druart C. Bindels L. B. et al. (2013). Cross-talk between *Akkermansia muciniphila* and intestinal epithelium controls diet-induced obesity. *Proc. Natl. Acad. Sci. U. S. A.* 110 9066–9071. 10.1073/pnas.1219451110 23671105 PMC3670398

[B15] Everson C. A. Toth L. A. (2000). Systemic bacterial invasion induced by sleep deprivation. *Am. J. Physiol. Regul. Integr. Comp. Physiol.* 278 R905–R916. 10.1152/ajpregu.2000.278.4.R905 10749778

[B16] Galli S. J. Tsai M. Piliponsky A. M. (2008). The development of allergic inflammation. *Nature* 454 445–454. 10.1038/nature07204 18650915 PMC3573758

[B17] Ganesh B. P. Klopfleisch R. Loh G. Blaut M. (2013). Commensal *Akkermansia muciniphila* exacerbates gut inflammation in *Salmonella typhimurium*-infected gnotobiotic mice. *PLoS One* 8:e74963. 10.1371/journal.pone.0074963 24040367 PMC3769299

[B18] Göker M. Gronow S. Zeytun A. Nolan M. Lucas S. Lapidus A. et al. (2011). Complete genome sequence of *Odoribacter splanchnicus* type strain (1651/6). *Stand. Genomic Sci.* 4 200–209. 10.4056/sigs.1714269 21677857 PMC3111987

[B19] Ivanov I. I. Atarashi K. Manel N. Brodie E. L. Shima T. Karaoz U. et al. (2009). Induction of intestinal Th17 cells by segmented filamentous bacteria. *Cell.* 139 485–498. 10.1016/j.cell.2009.09.033 19836068 PMC2796826

[B20] Iwakiri Y. Sampson D. A. Allen K. G. (2002). Suppression of cyclooxygenase-2 and inducible nitric oxide synthase expression by conjugated linoleic acid in murine macrophages. *Prostaglandins Leukot Essent Fatty Acids* 67 435–443. 10.1054/plef.2002.0454 12468265

[B21] Kim W. R. Flamm S. L. Di Bisceglie A. M. Bodenheimer H. C. (2008). Public policy committee of the american association for the study of liver disease. serum activity of alanine aminotransferase (ALT) as an indicator of health and disease. *Hepatology* 47 1363–1370. 10.1002/hep.22109 18366115

[B22] Kishino S. Takeuchi M. Park S. B. Hirata A. Kitamura N. Kunisawa J. et al. (2013). Polyunsaturated fatty acid saturation by gut lactic acid bacteria affecting host lipid composition. *Proc. Natl. Acad. Sci. U. S. A.* 110 17808–17813. 10.1073/pnas.1312937110 24127592 PMC3816446

[B23] Lamas B. Richard M. L. Leducq V. Pham H. P. Michel M. L. Da Costa G. et al. (2016). CARD9 impacts colitis by altering gut microbiota metabolism of tryptophan into aryl hydrocarbon receptor ligands. *Nat. Med.* 22 598–605. 10.1038/nm.4102 27158904 PMC5087285

[B24] Laursen M. F. Sakanaka M. von Burg N. Mörbe U. Andersen D. Moll J. M. et al. (2021). Bifidobacterium species associated with breastfeeding produce aromatic lactic acids in the infant gut. *Nat Microbiol.* 6 1367–1382. 10.1038/s41564-021-00970-4 34675385 PMC8556157

[B25] Machado R. B. Hipólide D. C. Benedito-Silva A. A. Tufik S. (2004). Sleep deprivation induced by the modified multiple platform technique: quantification of sleep loss and recovery. *Brain Res.* 1004 45–51. 10.1016/j.brainres.2004.01.019 15033418

[B26] Miao M. Wang Q. Wang X. Fan C. Luan T. Yan L. et al. (2022). The protective effects of inulin-type fructans against high-fat/sucrose diet-induced gestational diabetes mice in association with gut microbiota regulation. *Front. Microbiol.* 13:832151. 10.3389/fmicb.2022.832151 35495651 PMC9048744

[B27] Poroyko V. A. Carreras A. Khalyfa A. Khalyfa A. A. Peris E. Chang E. et al. (2016). Chronic sleep disruption alters gut microbiota, induces systemic and adipose tissue inflammation and insulin resistance in mice. *Sci. Rep.* 6:35405. 10.1038/srep35405 27739530 PMC5064361

[B28] Qu L. Ren J. Huang L. Pang B. Liu X. Liu X. et al. (2018). Antidiabetic effects of *Lactobacillus casei* fermented yogurt through reshaping gut microbiota structure in type 2 diabetic rats. *J. Agric. Food Chem.* 66 12696–12705. 10.1021/acs.jafc.8b04874 30398060

[B29] Ricciotti E. FitzGerald G. A. (2011). Prostaglandins and inflammation. *Arterioscler. Thromb. Vasc. Biol.* 31 986–1000. 10.1161/ATVBAHA.110.207449 21508345 PMC3081099

[B30] Ridlon J. M. Kang D. J. Hylemon P. B. Bajaj J. S. (2014). Bile acids and the gut microbiome. *Curr. Opin. Gastroenterol.* 30 332–338. 10.1097/MOG.0000000000000057 24625896 PMC4215539

[B31] Roager H. M. Licht T. R. (2018). Microbial tryptophan catabolites in health and disease. *Nat. Commun.* 9:3294. 10.1038/s41467-018-05470-4 30120222 PMC6098093

[B32] Schaffer S. Kim H. W. (2018). Effects and mechanisms of taurine as a therapeutic agent. *Biomol. Ther.* 26 225–241. 10.4062/biomolther.2017.251 29631391 PMC5933890

[B33] Schroeder B. O. Bäckhed F. (2016). Signals from the gut microbiota to distant organs in physiology and disease. *Nat. Med.* 22 1079–1089. 10.1038/nm.4185 27711063

[B34] Singh R. K. Gupta S. Dastidar S. Ray A. (2010). Cysteinyl leukotrienes and their receptors: molecular and functional characteristics. *Pharmacology* 85 336–349. 10.1159/000312669 20516735

[B35] Smith P. M. Howitt M. R. Panikov N. Michaud M. Gallini C. A. Bohlooly-Y M. et al. (2013). The microbial metabolites, short-chain fatty acids, regulate colonic Treg cell homeostasis. *Science* 341 569–573. 10.1126/science.1241165 23828891 PMC3807819

[B36] Suchecki D. Duarte Palma B. Tufik S. (2000). Sleep rebound in animals deprived of paradoxical sleep by the modified multiple platform method. *Brain Res.* 875 14–22. 10.1016/s0006-8993(00)02531-2 10967294

[B37] Tripathi A. Debelius J. Brenner D. A. Karin M. Loomba R. Schnabl B. et al. (2018). The gut-liver axis and the intersection with the microbiome. *Nat. Rev. Gastroenterol. Hepatol.* 15 397–411. 10.1038/s41575-018-0011-z 29748586 PMC6319369

[B38] Wahlström A. Sayin S. I. Marschall H. U. Bäckhed F. (2016). Intestinal crosstalk between bile acids and microbiota and its impact on host metabolism. *Cell Metab.* 24 41–50. 10.1016/j.cmet.2016.05.005 27320064

[B39] Wang J. Ren Y. Chen J. Chen S. Li X. Chen J. et al. (2025). *Bifidobacterium animalis* subsp. Lactis A6 alleviates comorbid constipation and depression by rebalancing tryptophan metabolism. *Sci. Bull.* 70 3738–3742. 10.1016/j.scib.2025.04.042 40328604

[B40] Williams B. B. Van Benschoten A. H. Cimermancic P. Donia M. S. Zimmermann M. Taketani M. et al. (2014). Discovery and characterization of gut microbiota decarboxylases that can produce the neurotransmitter tryptamine. *Cell Host Microbe* 16 495–503. 10.1016/j.chom.2014.09.001 25263219 PMC4260654

[B41] Zelante T. Iannitti R. G. Cunha C. Giovannini G. Pieraccini G. Zecchi R. et al. (2013). Tryptophan catabolites from microbiota engage aryl hydrocarbon receptor and balance mucosal reactivity via interleukin-22. *Immunity* 39 372–385. 10.1016/j.immuni.2013.08.003 23973224

